# Muriate of Cocaine

**Published:** 1884-11

**Authors:** 


### Muriate of Cocaine.

For some time laryngologists have used this substance to produce a local insensibility of the vocal cords. Dr. Carl Kollar, a young physician of Vienna, knowing this, was led to experiment with it upon the conjunctiva, beginning upon animals and afterwards testing its effects upon himself and other men. The results of his experiments were presented to the Ophthalmological Congress of Heidelburg in a short paper read before that body September 15th. The *Ophthalmic Review* (London), gives the following summary: "One or two drops of a two per cent. solution instilled into the eye produced a slight sensation of burning; in two minutes the cornea and conjunctiva become partially anæsthetic, and completely so in from ten to fifteen minutes, and this lasts for ten minutes. The drug causes a slight mydriasis, which lasts a couple of hours, and is accompanied by some paresis of accommodation, but reaction to light and eserine is not obliterated." He stated that during the anæsthesia the anterior surface of the globe can be cut, torn, scratched or rubbed without sensation. This discovery was made by Dr. Kollar only two weeks before the time of reading his paper at the meeting of the congress.

The wonderful and astonishing effects claimed for this drug can only be equalled by those of opium and general anæsthetics. It may not only be utilized in diseases and operations upon the eye, but also in the ear and upon mucous surfaces generally for similar purposes. Dr. Hubbell, of this city, who is one of the first to test its properties, will give the results of his experiments to the readers of the Journal at an early date.

